# Investigating mechanism of the effect of emotional facial expressions on attentional processing by data clustering approach

**DOI:** 10.1038/s41598-023-33197-w

**Published:** 2023-04-18

**Authors:** Yuezhi Li, Weifeng Zhao, Xiaobo Peng

**Affiliations:** 1 College of Mechatronics and Control Engineering, Shenzhen University, Shenzhen, 518060 Guangdong China; 2 Department of Psychiatry, Shenzhen Nanshan People’s Hospital and the 6th Affiliated Hospital of Shenzhen University Health Science Center, Shenzhen, 518052 Guangdong China

**Keywords:** Biomarkers, Neurophysiology

## Abstract

To explore the mechanism of the effect of emotional facial expression on attentional process, time course and topographic map of Electroencephalographic activities affected by emotional stimuli were investigated. Emotional Stroop task was used to collect 64-channel event-related potentials (ERP) in nonclinical participants, and data clustering was applied to find significant effect of sad and happy facial expression on ERP. Several significant ERP clusters were found in the sad and happy conditions respectively. In the sad condition, the decreased N170 in the bilateral parietooccipital areas, the increased P3 in the right centroparietal region and the increased negative deflection between 600 and 650 ms in the prefrontal regions were observed, these alterations reflected inhibited perceptual processing of sad facial expression, and increased activations of the orienting network and the executive control network in attentional system, respectively. In the happy condition, increased negative slow wave was found in the left centroparietal region indicating strengthened awareness and readiness for successive trials. Importantly, nonpathological attentional bias to sad facial expression in nonclinical participants was associated with inhibited perceptual processing and increased activations of the orienting and executive control networks. It provides the basis for better understanding and application of attentional bias in psychiatric clinical utilization.

## Introduction

The emotional Stroop task (EST) has been widely used to assess the extent to which emotional stimuli captured attentional resources. It was generally suggested that disproportionately more processing resources were attracted by emotional stimuli due to the activation of specific emotion-related knowledge structures. Therefore, during the task, interference may arise from the emotional material that activated task irrelevant, self-preoccupying processes and consume attentional capacity, and on the other hand, interference may be generated from the greater cognitive effort that is required to block out the perception of negative stimuli and render such stimuli unconscious^1^.

Attentional bias (AB) for emotional stimuli is a central feature of many cognitive theories of psychopathology. In psychopathology, emotional disturbance resulted in emotional stimuli becoming more salient, therefore biased the estimate of the danger or harm, and subsequently further increased emotional disturbance. The use of the EST demonstrated that AB existed in various mental disorders, such as the attentional maintenance time of negative facial expressions in patients with depression was longer than that in healthy controls, and the reaction time of bipolar disorder patients was slower than controls in response to emotional stimuli, and patients with generalized anxiety disorder were slower than non-anxious controls at color naming both threat words and positive words^2–7^, and there was evidence for an AB towards generally negative emotional words in patients with borderline personality disorder compared to nonclinical participants. Furthermore, AB was suggested to be sensitive to the differences between types of psychopathology^7–9^, such as there was evidence for an AB towards negative emotional stimuli in patients with borderline personality disorder than patients with anxiety and depression. In addition, the ability of participants to override AB in some environments was investigated, and such an override was found in nonclinical participants, whereas it had not been observed in clinical patient groups^10^. When AB can be overridden, it indicated that nonclinical participants were able to expend extra effort to exit the vicious cycle of emotional disturbance causing AB^11^.

Precise understanding the mechanism of AB in patients is important for clinical utilizations, and studying the nonpathological AB or how the emotional interference is overridden in nonclinical participants provides a basis. In a large number of studies with EST, behavioral data including accurate rate, reaction time, saccade, etc. has been widely used to demonstrate emotional effect indicating AB or override of AB. In comparison, Electroencephalographic (EEG) activities modulated by emotional Stroop interference has been less studied. In some event-related potentials (ERP) studies, emotional faces were used as distractor stimuli to find emotional Stroop interference in nonclinical participants, however, their findings were still inconsistent. Regarding the early P1 component, findings were mixed (3 reported emotion effects, 7 not). For the N170, seven studies reported emotion effects and six studies did not. Regarding the late ERP components, only a few studies reported emotional modulations of the Early Posterior Negativity (EPN) (4 yes, 1 no) or P3/ Late Positive Potential (LPP) (4 yes, 2 no)^12,13^. Together, the EEG spatiotemporal dynamics modulated by AB and override of AB are still not well known even in nonclinical participants. We suggest that the spatiotemporal property of EEG in the conditions of AB and override of AB should be investigated by the most appropriate data analysis method respectively.

To find out the spatiotemporal dynamics of involved neuronal structure in EST, AB or override of AB needs to be investigated by event-related brain potentials (ERP) across the brain. Then, the ERP results should be incorporated to an attention model that outlines the attentional processes and neural structure to find different attentional processing. An attentional model presented by literature^14^ may be such a model, offering an opportunity for better understanding how emotional stimuli modulate different attentional processing. In the model, the attention system was divided into three networks, each representing a different set of attentional processes. The attention framework included an alerting network that focused on brain stem arousal systems along with right hemisphere systems related to sustained vigilance; an orienting network that focused on, among other regions, parietal cortex; and an executive network which included cingulo-opercular and frontoparietal control systems.

In this study, an EST was used and the entire time course of ERP at dense scalp sites was examined. To address multiple comparisons problems, data clustering in combination with permutation tests was applied to find significant effect. The permutation tests produced so-called significant clusters in a two-dimensional plane formed by the dimensions space and time, and could be used to drastically increase the sensitivity of statistical tests for this type of data. This idea has led to the development of cluster-based permutation tests^15^. Because this proposed data-driven approach was applied to all electrodes and all time points, which had the potential to fully characterize the spatiotemporal properties of the ERP response while avoiding the constraints of an arbitrary selection of specific electrode sites or time periods, we believed that spatiotemporal dynamics of the emotional Stroop interference could be accurately represented.

We hypothesized that nonpathological AB might occur in nonclinical participants in this experiment, and the proposed study aimed to find the mechanism of nonpathological AB in the emotional Stroop processing, forming the basis for further study of EEG activity regulated by AB in various mental disorders.

## Materials and methods

### Participants

Thirty two healthy undergraduate dextromanual students (6:4 male to female, and mean 20.5 years) were recruited to participant in the EST. This study was performed in accordance with relevant guidelines and regulations approved by the institutional review board of Shenzhen University and written informed consent was obtained from each participant before the experiment. These participants hadn’t taken any medicine before, and no personal or family history of psychiatric or neurological disease. In addition, informed consent for publication of identifying information/images in an online open-access publication was obtained.

### Experiment procedure

The emotional Stroop task consisted of 40 neutral, 40 sad and 40 happy facial pictures, which were extracted from the Chinese Facial Affective Picture system (CFAPS)^16^. The 120 facial pictures had been assessed for its valence and arousal on a 9-point scale by another 100 Chinese adults in a previous work. The ANOVA performed on the average scores of the 120 faces in the previous work showed that the three categories of faces have significantly different emotional valence scores (F(2,117) = 285.2, p < 0.001, happy = 6.24 ± 0.12, sad = 3.06 ± 0.23, neutral = 4.20 ± 0.12) as well as arousal scores (F(2,117) = 87.6, p < 0.001, happy = 5.78 ± 0.21, sad = 6.34 ± 0.37, neutral = 3.60 ± 0.21).

**Figure 1:**
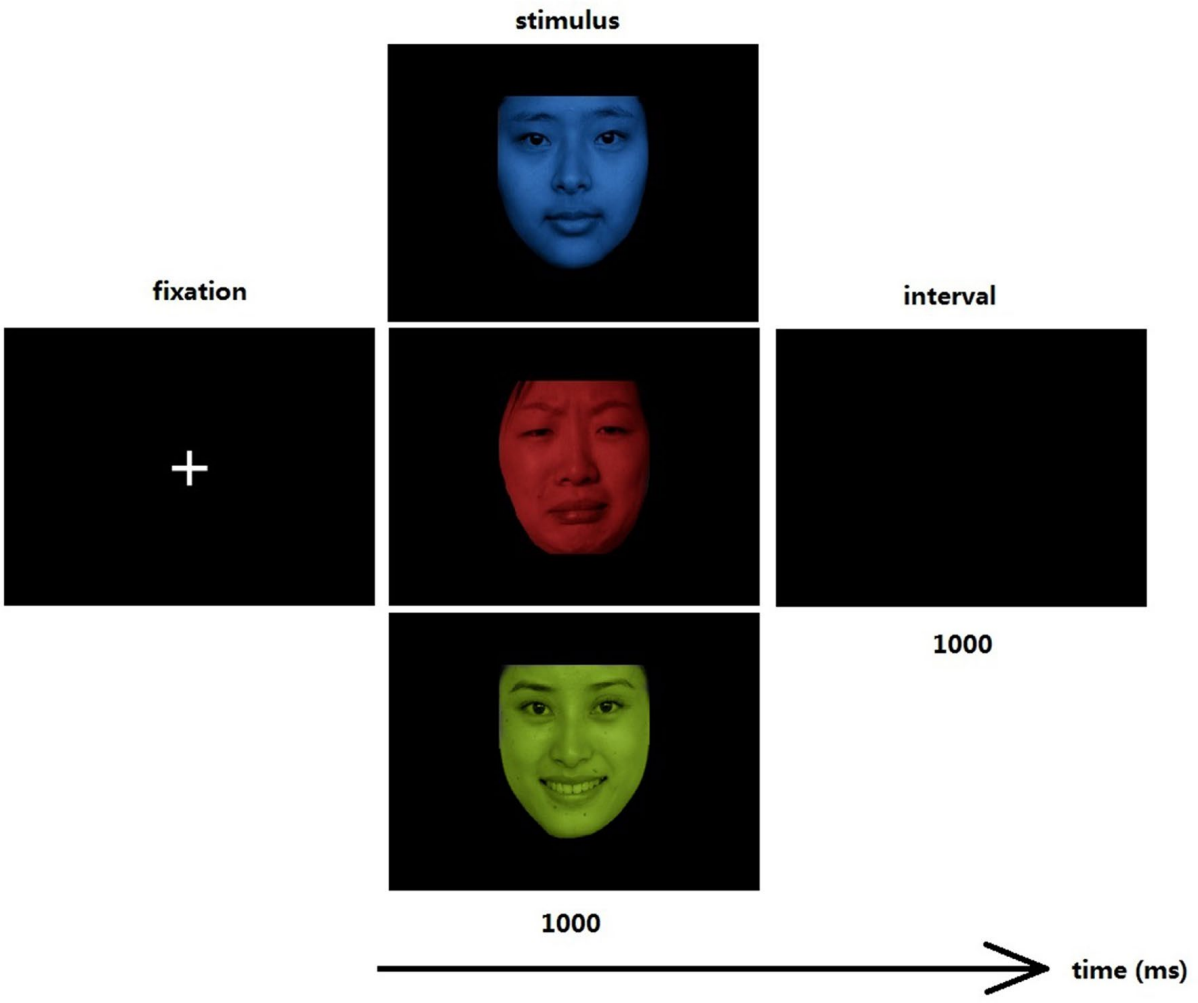
Illustration of one trial in this study. Participants watched 1000 ms of stimulus in the form of neutral, sad or happy face, separated by an inter-stimulus interval of 1000 ms.

Each facial picture was made into red, green or blue using drawing software. Participants were instructed to identify and nominate the color of the face by pressing a button as soon as it appeared. They should press 1 on the keypad with their index finger if the face was red, 2 with their middle finger if the face was green and 3 with their ring finger if the face was blue. Prior to experiments, participants were suitably familiarized with the keypad and when performing the tasks, they were asked to respond as quickly as possible but not at the expense of accuracy. Only these two components (accuracy and speed of responses) were emphasized and participants were not alerted to the affective component of the experiment. In the experiment, each face stimulus (sad, happy or neutral) appeared for 1000 ms separated by an inter-stimulus interval of 1000 ms (Fig. 1). The task included six blocks, with 40 face stimuli presented per block of 80 s, and the facial expressions and colors for each face stimulus were randomly presented. Before the task, all participants underwent a practice block of 20 stimuli.

### EEG recording and analysis

The EEG data were recorded with the BrainAmp amplifier (Brain Products, Munich, Germany) and Braincap electrode cap (EASYCAP, Herrsching, Germany). 64 Ag/AgCl electrodes were placed according to the 10–20 System and intermediate sites. All channels were referenced during recording to electrode (FCz) with a forehead ground (AFz). To remove eye movements, an additional electrode, Iz, was placed on the infraorbital ridge of the right eye to record the vertical electrooculogram. Electrode impedance was maintained below 5 kΩ throughout the experiment. The EEG and electrooculogram were recorded without filtering, and digitized at a sampling rate of 1000 Hz^17^.

The EEG was digitally filtered with a band-pass filter of 0.16–30 Hz (24 dB/Octave), Electrooculogram artifacts were corrected by ocular correction using the independent components analysis algorithm in Brain Vision Analyzer software (Brain Products). EEG data referenced to FCz were recalculated against the average reference, and a time epoch for each event of 1600 ms (200 ms pre-stimulus and 1400 ms post-stimulus) was used. For each epoch, a baseline correction was performed according to the data 200 ms prior to the stimulus. To avoid eye movement and other artifacts, all epochs exceeding ± 100 μV in any channel were excluded from average analysis^18^. The number of accepted epochs was 67.3 ± 2.5, 66.4 ± 3.2 and 67.6 ± 2.9 for neutral, sad, and happy conditions, respectively. The number of accepted trials did not differ significantly across conditions (ps > 0.05). The averaged ERP were computed separately for the neutral, sad, and happy conditions. Grand average difference waveforms were obtained from the average of all participants.

### Statistical analysis

64 channels of ERP data between the happy/sad and the neutral conditions, were compared with permutation-based cluster analysis as implemented in BESA Statistics (v2.0, BESA Software). The main idea is that if a statistical effect is found over an extended time period in several neighboring electrodes, it is unlikely that this effect occurs by chance.

In the first step, two-tailed t-tests were calculated for all data points of ERP to obtain preliminary significant effects between conditions. The ERPs data points for t-tests were from 0 to 1400 ms. Then, we selected all data points according to alpha setting of p < 0.05 and clustered the selected data points (electrode, time) on the basis of temporal and spatial adjacency. In this study, because circle arc length between two neighboring electrodes on Braincap electrode cap was less than 4 cm, we considered electrodes to be neighbors if their distance is less than 4 cm. As a result, the preliminary significant clusters were obtained and sum of t-values within each cluster was calculated.

In the second step, a random permutation test was calculated 1000 times. In each permutation test, the data of the two conditions were interchanged randomly for each participant, and t-tests were calculated for all data points. At the end of each permutation test, *t* values of adjacent data points (electrode, time) that fell below a *p-*value of 0.05 were summed and the cluster with the highest sum of *t* values was kept. By these means, a null distribution of cluster sums was created from the 1000 permutation tests. Based on this distribution, the *p*-value of the preliminary cluster was estimated^15,19–21^. In addition, a cluster could be identified as positive or negative, depending on the direction of its statistical effect.

### Ethical approval

This study was approved by the institutional review board of Shenzhen University. Permission from all participants for both study participation and publication of identifying information/images in an online open-access publication was obtained.

## Results

### Behavioral data

**Figure 2:**
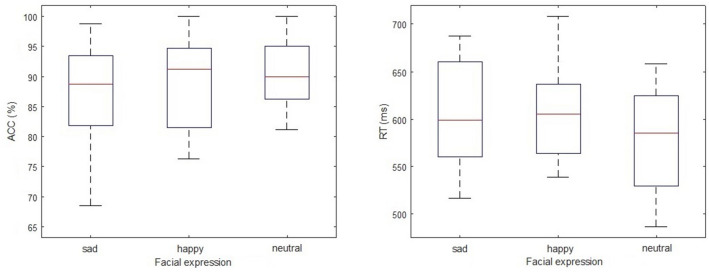
Boxplots of ACC and RT in each condition in EST. The top and bottom lines of the plot indicates the maximum and minimum values, the top and bottom lines of the box indicates the 75th and 25th percentile values, and the red line indicates the median value.

The accurate rate (ACC) and reaction time (RT) in each condition were illustrated for 32 participants (Table 1; Fig. 2). Mean of ACC and RT for all participants were analyzed by repeated measures ANOVAs, with emotional facial expressions (Sad, Happy, Neutral) as within-subject factor. A repeated measures ANOVA revealed a significant main effect of emotional facial expressions on RT (F = 9.174, P < 0.001). Post-hoc analysis revealed that RT in the neutral condition was significantly shorter than that in both the sad condition (p < 0.001) and the happy condition (p < 0.01). Another repeated measures ANOVA did not reveal significant main effect of emotional facial expressions on ACC (F = 2.766, P = 0.068).

**Table 1:** RT and ACC in the three conditions in EST (mean ± SD, n = 32).

	Neutral condition	Sad condition	Happy condition
ACC (%)	90.3 ± 6.1	87.0 ± 9.0	89.4 ± 7.7
RT (ms)	574.9 ± 58.2	606.0 ± 56.0	605.4 ± 49.2

### ERP data

**Figure 3:**
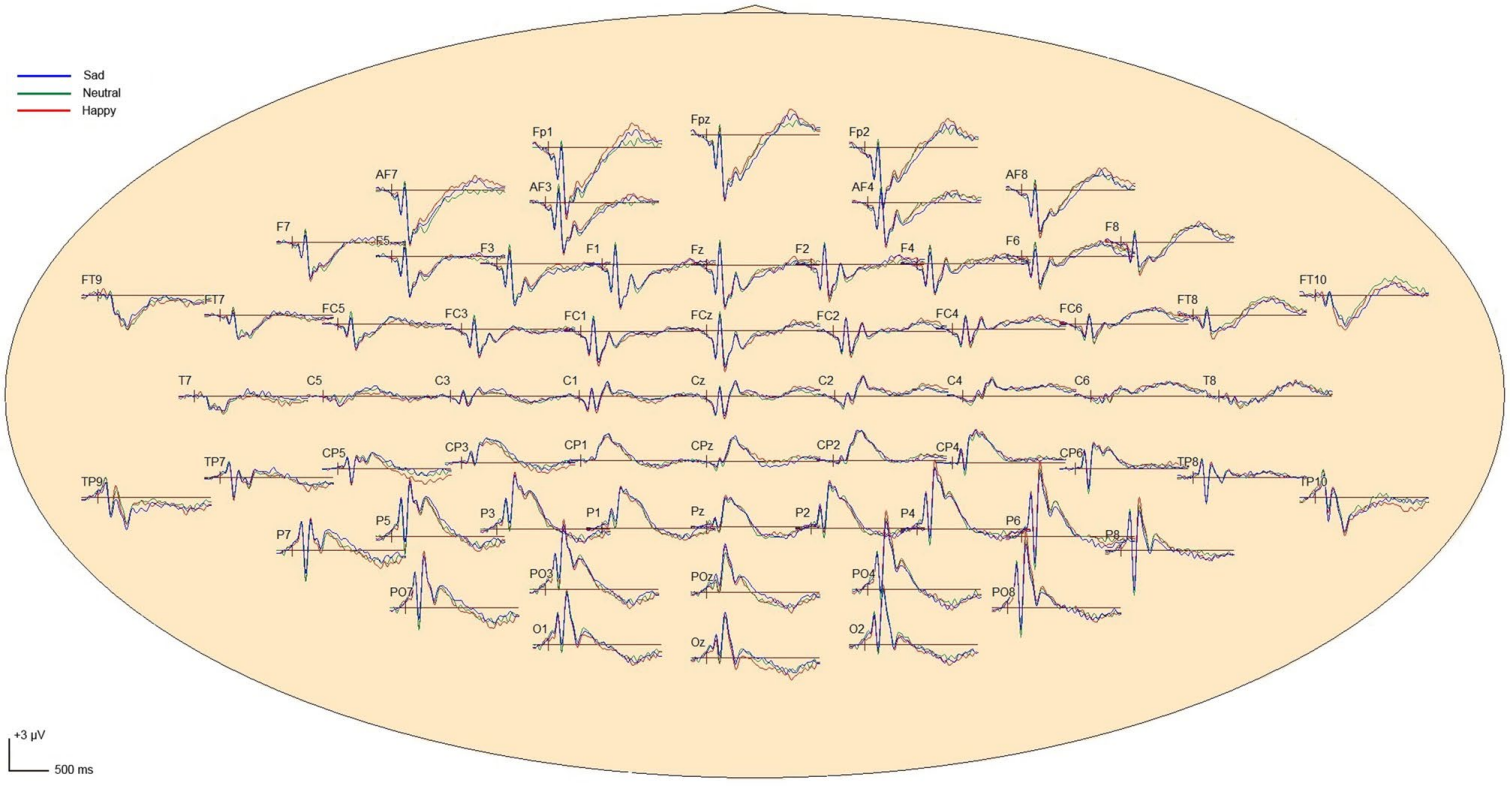
Top view of 64-channel ERP waveforms in the sad, happy and neutral conditions. Blue curves present grand average ERP waveforms across all participants in the sad condition; green curves present grand average ERP waveforms across all participants in the neutral condition; red curves present those in the happy condition.

**Figure 4:**
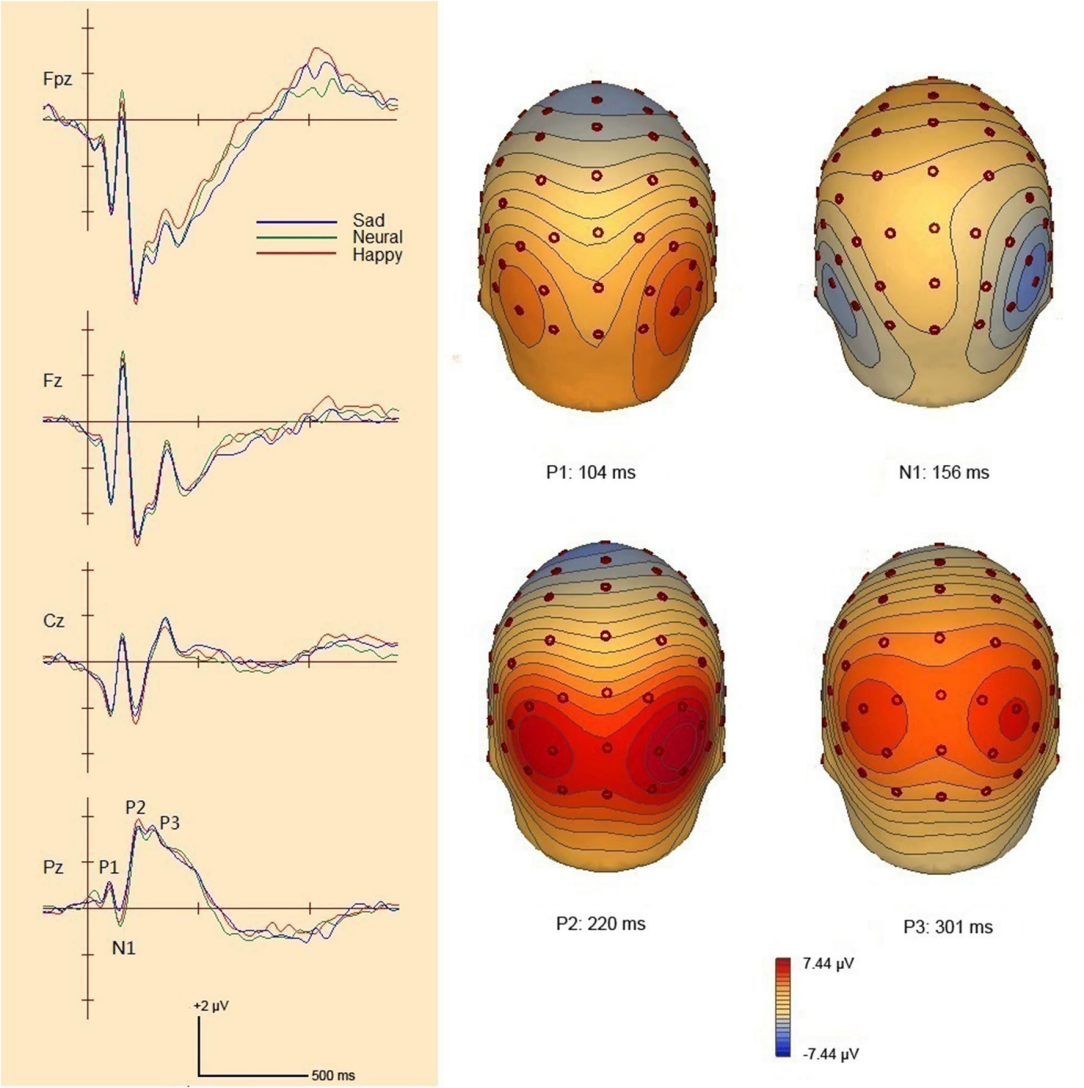
ERP waveforms at typical electrode locations (left panel) and scalp voltage maps of ERP components in the neutral condition (right panel).

The averaged ERP waveforms in each condition were shown in Fig. 3, and the ERP waveforms at typical electrodes and the scalp voltage maps in the neutral condition were shown in Fig. 4. ERP for targets presented a positive component P1 over parieto-occipital areas, a negative component N1 over bilateral ventral parietal areas, a positive deflection P2 over parieto-occipital areas, and a positive component P3 over parietal areas.

### ERP difference

**Figure 5:**
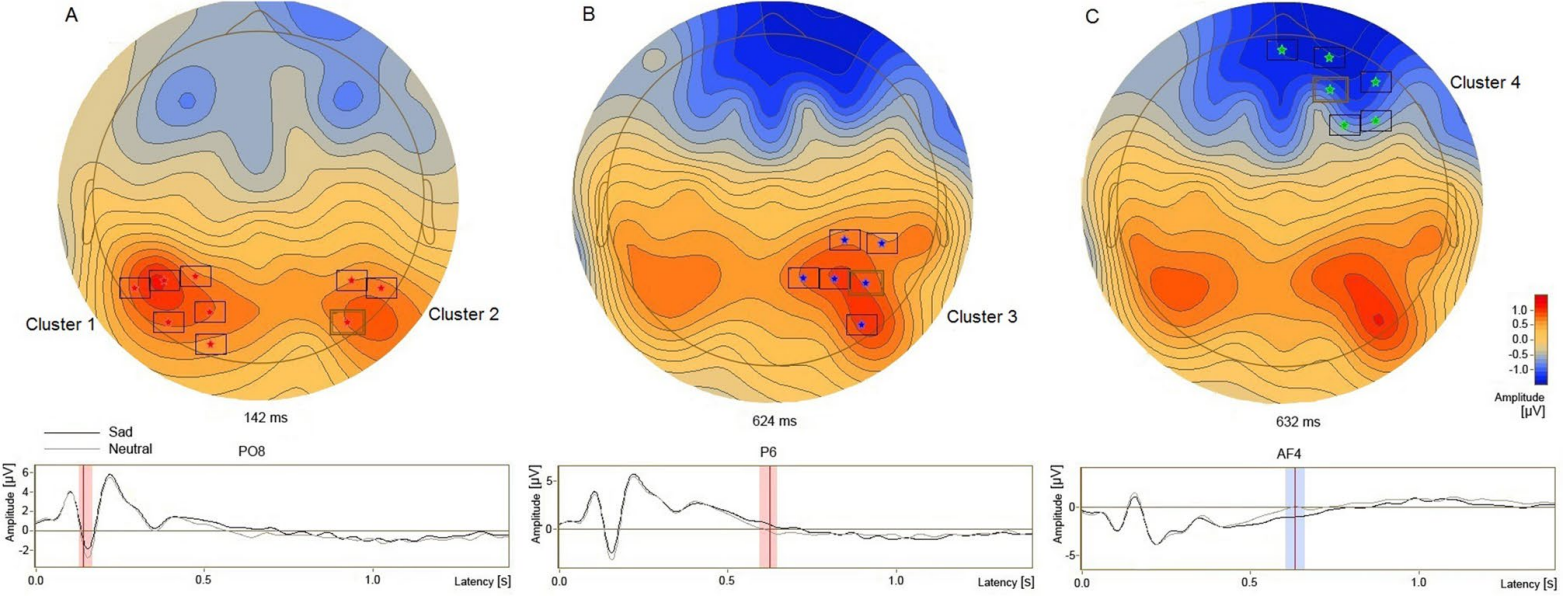
Significant clusters obtained from comparison between the sad condition and the neutral condition. The electrodes in the significant clusters were marked with asterisks and boxes and the differences in scalp voltages between the two conditions were shown in the upper panel. The low panel showed the ERP waveforms of typical electrodes PO8, P6 and AF4 in the significant clusters in the two conditions, and the time periods when the ERP amplitudes were significantly different between conditions.

Significant clusters obtained from comparison between the sad and the neutral conditions were shown in Fig. 5, with four clusters being observed. In a period at around 142 ms, a lower N1 was found in the sad condition than in the neutral condition in the bilateral parietooccipital areas (Fig. 5A; Cluster 1 and 2; p = 0.007). In a period at around 624 ms, a higher P300 deflection was identified in the sad condition than in the neutral condition in the right centroparietal area (Fig. 5B; Cluster 3; p = 0.008). Additionally, in a period at around 632 ms, a larger negative deflection was found in the sad condition than in the neutral condition in the midline and right prefrontal areas (Fig. 5C; Cluster 4; p = 0.018).

**Figure 6:**
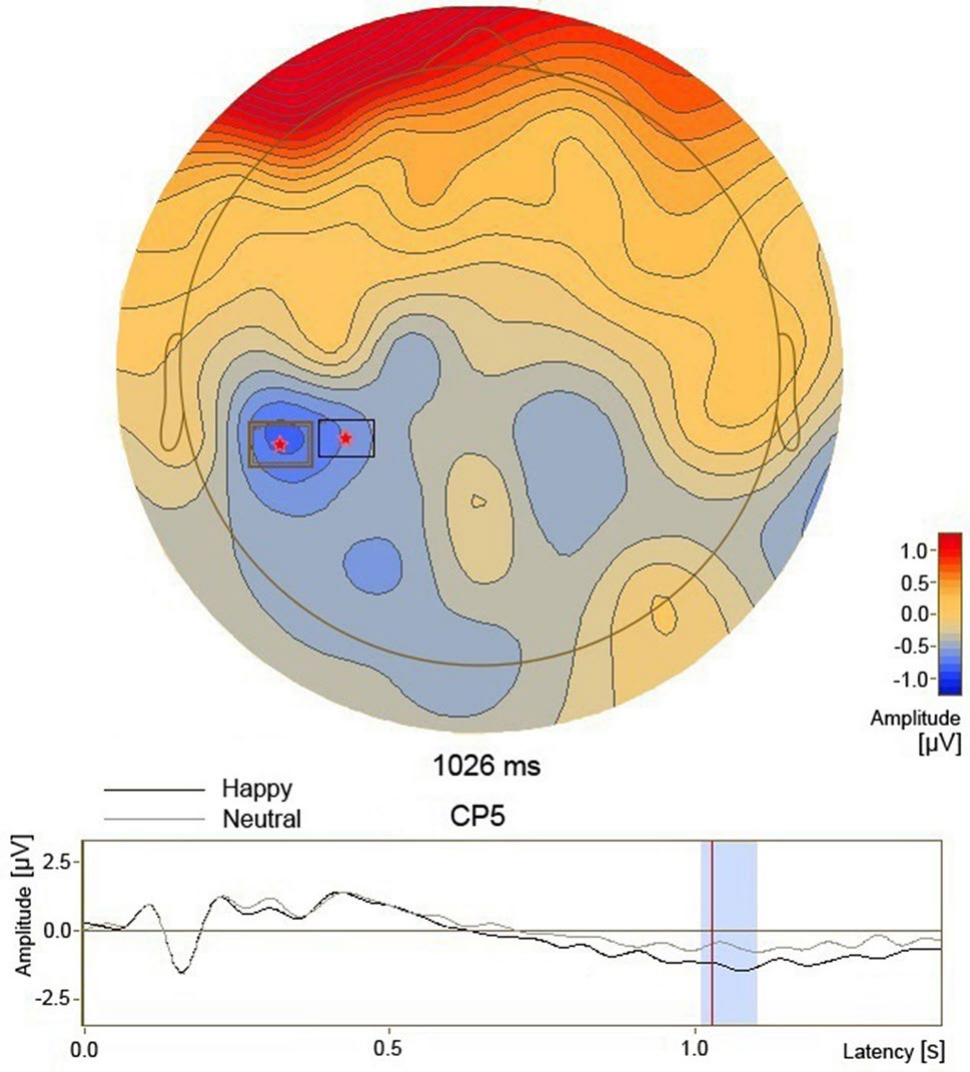
Significant cluster obtained from comparison between the happy condition and the neutral condition. The electrodes in the significant cluster were marked with asterisks and boxes and the differences in scalp voltages between the two conditions were shown in the upper panel. The low panel showed the ERP waveforms of typical electrode CP5 in the significant cluster in the two conditions, and the time period when the ERP amplitudes were significantly different between conditions.

Significant clusters obtained from comparison between the happy and the neutral conditions were shown in Fig. 6, and only one significant cluster was observed. In the period at around 1026 ms, a larger negative deflection was found in the happy condition than in the neutral condition mainly in the left centroparietal area (p = 0.023).

## Discussion

In this EST, four significant ERP alterations were found in the condition of sad facial expression using parametric t-tests followed by data clustering and permutation tests. In the early attentional processing at about 142 ms, the N1 component in the sad condition was lower than that in the neutral condition in the bilateral parietooccipital areas. In the late attentional processing at around 624 ms, the P3 component in the sad condition was higher than that in the neutral condition in the right centroparietal region. In the period at around 632 ms, a negative deflection in the sad condition was larger than that in the neutral condition in the midline and right prefrontal regions. In the happy condition, one significant ERP alteration was found. In the period at around 1026 ms, a negative deflection was larger than that in the neutral condition in the left centroparietal region.

As described in the literature^14^, the attention system included an alerting network related to sustained vigilance; an orienting network that focused on parietal cortex; and an executive control network, which included midline frontal/anterior cingulate cortex.

In the orienting network, a dorsal attention system and a ventral attention system were related to orienting to stimuli. The dorsal system focused on the interparietal sulcus and the frontal eye field was associated with rapid strategic control of attention, while the ventral system focused on the temporoparietal junction and ventral frontal cortex was associated with breaking of attention and switch.

In the executive control network, due to the limited capacity of the attention system, the moment of target detection and awareness of the target produced interference across the system and had often been called focal attention. The activity found in the medial frontal/anterior cingulate was involved in this process^22–24^. And this activity sustained across the trials of the task, was related to the maintenance of task parameters/top-down control and performance feedback^14,25,26^.

In the present sad condition, it could be speculated that the participants would frequently break their attention focus on sad facial expression and switch to color naming, which resulted in increased regional activity related to orienting network in the sad condition compared to the neutral condition. Therefore, our result suggested that the higher P3 deflection found in the right parietal region in the sad condition might be associated with increased activity in the orienting network for rapid control of attention and attention switching.

At the same time, when the executive control network was activated by color naming, the moment of target detection produced interference across the attention system. It could be speculated that because more attentional resources were allocated to sad facial expression in the sad condition compared to the neutral condition, extra efforts were required to capture awareness of the target. As it was generally accepted that the medial frontal cortex and anterior cingulate (MFC/ACC) were involved in executive control, the result suggested that the larger negative deflection in the period at around 632 ms in the prefrontal regions indicated the extra activation in MFC/ACC in the sad condition. In addition, there might be two separate executive networks acting independently in the attention system^14^. Besides the cingulo-opercular control network, MFC/ACC, which showed maintenance across trials and acted as stable background maintenance for task performance, the other control network, the frontoparietal system, in contrast, was thought to be related to task switching and initiation and to adjustments within trials in real time. This frontoparietal system provided another explanation for the higher P3 in the right parietal region in the sad condition. These two explanations may not be contradictory since previous imaging evidence showed that the orienting and frontoparietal executive networks were separate in adulthood, they may have a common origin.

The N170 component was identified in plenty of face processing tasks^27–29^, and the superior temporal gyrus (STG) was suggested to be related to the perceptual processing of faces, such as expression or view^30,31^. Sources located in or around this region and directed towards the lateral part of the cortex might also contribute, together with the fusiform gyrus to the N170^32–34^. In the present study, difference of the N170 between the sad and neutral conditions, located in/around the bilateral scalp temporoparietal areas, was consistent with previous findings. Because the resultant clusters and the difference ERP waveforms (Fig. 5) both indicate a decrease of the N170 in the sad condition in this study, inconsistent with an increase in some studies^12^, we suggested that the early perceptual processing of sad faces was inhibited as some other studies^34,35^.

Besides the N170 component, the Early Posterior Negativity (EPN) reflected differences between emotionally arousing and neutral picture contents, and it emerged early between 200 and 300 ms and in the temporo-occipital regions. Some previous studies had demonstrated EPN modulations by emotional facial expressions to suggest early perceptual processing^12^. However, such EPN effect was not observed in this study. The reason might be that we had used a more severe clustering method, and the effect was invalid unless it existed within a certain temporal and spatial neighborhood.

In the present happy condition, although no significant difference was found in the early perceptual and P3 processes, a larger negative deflection was found in the period at around 1026 ms in the left centroparietal region in the happy condition than in the neutral condition, and it indicated an enhanced negative slow wave (NSW) following the P3. As NSW would be a manifestation of anticipation or general readiness like the contigent negative variation (CNV)^36,37^, it was suggested that the increase of NSW reflected strengthened awareness and readiness for upcoming trial in the happy condition. This increased activity in the left centroparietal region in the happy condition might reflect a type of signal the happy emotional material increased CNV-related activity in midcingulate or left somatomotor cortex in participants^38,39^.

In this EST, significant effect of sad and happy facial expressions on RT existed indicating AB in nonclinical participants. It implicated that the participants did not override the effect of distraction from sad and happy facial expression by completely inhibiting perceptual processing of emotional expression and significantly increasing the task demand unit to name color. Together, the ERP spatiotemporal dynamics indicating inhibited early perceptual processing of sad faces, increased activations in the orienting network and the executive control network in attentional system specified the mechanism that underlies AB towards sad facial expressions in nonclinical participants. In addition, the ERP dynamics indicating strengthened awareness and readiness for successive trials might address the mechanism that underlies AB towards happy facial expressions. To best of our knowledge, no such finding on the mechanism underlying nonpathological AB has ever been reported.

## Conclusion and future directions

In the sad facial expression condition in EST, four significant ERP alterations were identified in nonclinical participants including the decreased N170 in the bilateral parietooccipital areas, the increased P3 in the right centroparietal regions and the increased negative deflection between 600 and 650 ms in the prefrontal regions. These alterations might reflect inhibited perceptual processing of sad facial expression and increased processing in the orienting network and the executive control networks in attentional system, respectively. In the happy facial expression condition in EST, increased NSW was found in the left centroparietal region indicating strengthened awareness and readiness for successive trials. In summary, the ERP responses indicating altered perceptual and attentional processing of sad facial expression specified the mechanism underlying nonpathological AB towards sad emotional stimuli, and the ERP responses indicating strengthened awareness and readiness for successive trials specified the mechanism underlying nonpathological AB towards happy emotional stimuli.

One limitation of this study is a relatively small sample size and a lack of pre-study sample size calculation. To calculate sample size, researchers must specify the variability of the outcome variable^40^, while it is very difficult to glean standard variance (SD) of ERP from similar studies currently.

In another ongoing study, we are examining the ERP and behavioral data in patients with depression using the same EST, significant difference is observed between patients and nonclinical participants (unpublished). Accordingly, in a future work, if the significant clusters between groups are used as feature vectors for pattern classification, it is possible to construct a classifier to distinguish patients with depression from healthy populations.

## Data Availability

The data that support the findings of this study are available from Shenzhen University, and the data are available from the authors upon reasonable request and with permission from Shenzhen University. Requests for materials should be addressed to Y.L. (email: yzli@szu.edu.cn).
